# “We are here too”: Experiences and perceived support needs of adolescent siblings of Paediatric oncology inpatients

**DOI:** 10.1111/bjhp.12785

**Published:** 2025-02-17

**Authors:** Rachel Batchelor, Matthew Hotton, Eloise Harris, Alex Lau‐Zhu, Annabel L. David

**Affiliations:** ^1^ The Oxford Institute of Clinical Psychology Training and Research University of Oxford Oxford UK; ^2^ Oxford Health NHS Foundation Trust Oxford UK; ^3^ Children's Psychological Medicine Oxford University Hospitals NHS Foundation Trust Oxford UK; ^4^ Department of Experimental Psychology University of Oxford Oxford UK; ^5^ Department of Brain Sciences Imperial College London London UK

**Keywords:** adolescents, cancer, hospital, oncology, paediatrics, psychosocial support, siblings

## Abstract

**Background:**

Adolescent siblings of children and young people (CYP) with cancer are at increased risk of psychosocial difficulties, yet many remain overlooked and unsupported. This project aimed to explore the experiences and perceived needs of adolescent siblings of paediatric oncology inpatients to inform service improvement recommendations for sibling support.

**Methods:**

Semi‐structured interviews were conducted with 10 siblings of CYP previously admitted to a paediatric oncology ward. Interviews were transcribed verbatim and analysed using reflexive thematic analysis. The findings were reviewed in consultation with staff and used to identify pragmatic/feasible recommendations for improving sibling support, organized using the three‐tier ‘Pediatric Psychosocial Preventative Health Model’ (PPPHM; Families, Systems & Health, 2006, 24, 381).

**Results:**

An overarching narrative of siblings wanting to feel part of the cancer journey was found, including their family's experience on the ward, with three key themes: (i) “what about me?”: overlooked and unseen, (ii) “always changing, never knowing”: the challenge of uncertainty and (iii) “let me be part of it all”: togetherness, communication and connection. These findings informed sibling support recommendations. Such recommendations included providing psychosocial screening, resources and opportunities for family time/communication and developmentally appropriate information to all siblings (universal support), monitoring psychosocial difficulties, siblings having someone to talk to and fostering family and peer connection for siblings requiring additional support (targeted support) and offering one‐to‐one psychological support and family therapy for persistent and/or escalating distress (clinical/treatment support).

**Conclusions:**

Based on the experiences of siblings, a range of sibling support recommendations have been identified. Implementation and evaluation of these recommendations are warranted.

**Statement of Contribution**

**What Is Already Known on this Subject?**

- Adolescent siblings of hospitalized children with cancer are at greater risk of psychosocial difficulties.
- The needs of siblings of children with cancer are often unmet, with support being inconsistent.
- Improving resources and perceptions can help families to manage stressors.

**What Does this Study Add?**

- This qualitative study explores the experiences and perceived support needs of adolescent siblings on a paediatric oncology ward to inform service improvement recommendations for sibling support.
- An overarching narrative was found of siblings wanting to feel part of their family's experience on the ward.
- Organized using the three‐tier ‘Pediatric Psychosocial Preventative Health Model’ (PPPHM; Kazak, 2006), recommendations are made to improve support for adolescent siblings on paediatric oncology wards, including screening for psychosocial difficulties, providing developmentally appropriate information and fostering family and peer connection.

## BACKGROUND

Approximately 1800 children and young people (CYP) aged 0–14 years old are diagnosed with cancer in the UK each year (Cancer Research UK, 2021b). Whilst survival rates are improving, a growing evidence base has drawn attention to adverse short‐ and long‐term psychosocial impacts experienced by CYP with cancer (Duran et al., 2020; Osmani et al., 2023). Such impacts of childhood cancer extend beyond the unwell child, affecting the daily routines and emotional balance of the wider family (Bates et al., 2021; Mu et al., 2015). Clinical guidelines acknowledge the importance of assessing and supporting the psychosocial needs of family members (National Institute for Health and Care Excellence [NICE], 2014). Additionally, the standards for the psychosocial care of children with cancer and their families proposed by Wiener et al. (2015) include a sibling psychosocial standard of care which, based on evidence, strongly recommends psychosocial services for siblings of CYP with cancer (Gerhardt et al., 2015). However, compared with CYP with cancer and their parents, siblings have remained largely overlooked, with support being inconsistent and needs often unmet (Davis, Alderfer, et al., 2023; Davis, Brosnan, et al., 2023).

Sibling relationships are characteristically distinct, often sharing a unique history and holding the potential for greater longevity than parental, spousal and peer relationships (Cicirelli, 2013; Noller, 2005). Siblings of CYP with cancer have been found to face increased risks of psychosocial difficulties, including anxiety, depression, posttraumatic stress symptoms and loneliness (Alderfer et al., 2010; Houtzager, 2004). Hospitalization can bring additional challenges to siblings, including routine disruptions, family separation and role adjustments (Niinomi & Fukui, 2022; Prchal & Landolt, 2012). Psychosocial difficulties have also been shown to intensify during hospitalizations, such as anxiety surrounding their unwell sibling's health (Nabors & Liddle, 2017).

Adolescent siblings of CYP with cancer have been found to experience more distress than younger siblings (Cordaro et al., 2012), yet little is known about their experiences of having a sibling in the hospital. Additionally, childhood cancer studies often combine siblings of diverse developmental stages (e.g. from young childhood to older adolescents) within the same sample, without specific consideration of the impact of the experience on each stage (Wilkins & Woodgate, 2005). Many have also used proxy informants, predominantly parents, despite known discrepancies between proxy and self‐reports (Pariseau et al., 2020; Paul et al., 2023; Schulte et al., 2016), highlighting the need for gathering perspectives of siblings directly.

Within family contexts, one theoretical model for understanding stress and coping is Hill's (1958) ABC‐X model. The model encompasses the elements of (A) stressor(s), (B) resources to cope with the stressor(s), (C) perceptions of the stressor(s), and (X) resulting level or likelihood of stress or crisis. The ABC‐X model posits that B and C determine if stressor(s) in category A result in crisis (greater levels of stress than the individual/family feel able to manage). For siblings of CYP with cancer, common stressors (A) may include diagnosis, bereavement and hospitalization; resources (B) evaluated as helpful include developmentally appropriate information, family support and peer support; and perceptions (C) that may be important include those surrounding the cancer, prognosis, family cohesion and their role (Batchelor, 2019; Incledon et al., 2015; Long et al., 2018; Zegaczewski et al., 2016). Therefore, supporting adolescent siblings of CYP with cancer with their resources and perceptions is important. One specific stressor for adolescent siblings that has not been sufficiently investigated is hospitalization of the CYP with cancer.

Understanding the experiences and perspectives of adolescent siblings of paediatric oncology inpatients could inform the development of practical service improvement recommendations to support with resources and perceptions and consequently foster psychosocial adjustment. With input from ward and oncology staff, such recommendations could then be organized using the ‘Pediatric Psychosocial Preventative Health Model’ (PPPHM; Kazak, 2006). The PPPHM outlines screening all families for indicators of higher psychosocial risks and then uses three tiers to guide support dependent on their level of need; ‘universal’ support available to all; ‘targeted’ additional support specific to needs and ‘clinical/support’ support for persistent and/or escalating distress. Therefore, considering feasibility, by providing low‐intensity resources (i.e. universally), fewer require more intensive support, as the psychosocial needs of more siblings can be met.

### Aims and key questions

This project aimed to identify the experiences and support needs of adolescent siblings when the CYP with cancer is on a paediatric oncology ward. Such findings could then inform the development of service improvement recommendations. Our two main improvement questions were:

1. What are the experiences and perceived needs of adolescent siblings of CYP during an inpatient stay for cancer?
2. What changes could be made to better support adolescent siblings of CYP on a paediatric oncology ward?

## METHODS

### Participants

The purposive sampling method was used with emails/letters, recruitment posters, and word‐of‐mouth to recruit adolescent siblings of CYP who had been admitted to a paediatric oncology ward.

The paediatric oncology ward being studied is based in the United Kingdom and has 13 beds for 0‐16‐year‐olds. Sibling support is currently limited. For example, activity days for siblings are infrequently accessed, especially by adolescents. Due to limited service provision, one‐to‐one psychological support is only offered in a minority of cases when families are extremely struggling (i.e. already reached crisis).

Eligibility criteria were (i) aged 11–16 years old and (ii) had a sibling who had stayed on the ward for at least 1 week in the previous 6 months. This ensured there had been a substantial inpatient stay since 17th May 2023, when siblings were allowed back on the ward following visiting restrictions imposed due to COVID‐19.

To minimize risks of distress, siblings were excluded from taking part if their sibling with cancer, or any other immediate family member with cancer, was currently an inpatient in any ward/hospital or receiving palliative care. Siblings were also excluded if their sibling with cancer had died, as support needs may differ among bereaved siblings (Foster et al., 2012; Ridley & Frache, 2020). Hospital staff screened families for eligibility. No siblings dropped out of the study following recruitment.

Ten siblings, all from different families, took part. To contextualize the data, background information was gathered for participating siblings (Table 1). Most siblings were White British, full siblings (of their sibling with cancer) and from nuclear families. Siblings were equally split in gender and position in the family (i.e. older, younger) compared to their sibling with cancer. Eight of the participating siblings had visited their sibling with cancer on the ward; two had not due to the distance of the hospital from home.

**TABLE 1:** Demographic and family‐related information of sibling participants.

Characteristic	Sibling without cancer (*N* = 10)
Age, mean (standard deviation; range)	14.10 (1.79; 11–16)
Age when a sibling was first diagnosed with cancer, mean (standard deviation; range)	11.80 (1.99; 9–14)
Gender	
Female	5
Male	5
Ethnicity, *n*	
White British	6
White Irish	1
White, Other	1
Black British	1
Chinese	1
Position in family (compared to sibling with cancer)	
Older	5
Younger	5
Number of siblings	
One	5
Two	4
Three	1
Relationship (to sibling with cancer)	
Full sibling	8
Half‐sibling	1
Stepsibling	1
Family composition	
Nuclear family	7
Single‐parent family	2
Stepfamily	1

Demographic and cancer‐related characteristics of their sibling with cancer were also gathered (Table 2). Most were White British and 60% were male. They covered a range of current ages, ages at diagnosis and types of common childhood/teenage cancer (Cancer Research UK, 2021a).

**TABLE 2:** Demographic and cancer‐related information of the siblings with cancer.

Characteristic	Sibling with cancer (*N* = 10)
Age, mean (standard deviation; range)	11.90 (3.03; 6–16)
Age at cancer diagnosis, mean (standard deviation; range)	9.40 (2.99; 4–14)
Gender	
Male	6
Female	4
Ethnicity, *n*	
White British	6
White Irish	1
White, Other	1
Black British	1
Chinese	1
Type of cancer, *n*	
Acute lymphocytic leukaemia (ALL)	2
Acute myeloid leukaemia (AML)	2
Osteosarcoma	2
Glioblastoma	1
Melanoma	1
Neuroblastoma	1
Wilms tumour (nephroblastoma)	1

### Lived/living experience involvement

In line with participatory research methodologies (Montreuil et al., 2021; Vaughn & Jacquez, 2020), three siblings (2 females, 1 male; aged 11–16 years‐old) were involved in developing the study. The study development period occurred prior to the recruitment period. Siblings were chosen who fit the eligibility criteria at the time of development (i.e. their sibling had been on the ward in the prior 6 months) but would likely not during the time of recruitment (as it would have been longer than 6 months since their sibling's latest ward admission). Therefore, the siblings who were involved in the development of the project were not the same siblings as those who participated.

The lived/living experience involvement included developing the main aims, interview schedule and project materials. Efforts were made (e.g. inclusive language, graphics representing different young people) to ensure the materials and questions were understandable, representative and applicable to a diverse range of siblings (e.g. age, gender, ethnicity, cancer type). They were offered payment in accordance with the service policy.

### Procedure

Due to being focused on evaluating sibling experiences on a specific ward only, this project was registered as a service evaluation under the National Health Service Trust where it was conducted, with approval obtained. The relevant clinical governance committee was then informed of the project's completion.

All siblings and their parent(s)/carer(s) were given a project information sheet. Siblings aged 11–15 provided written informed assent, with their parent/carer providing written informed consent, and siblings who were 16 provided their own written informed consent. Consent was rechecked verbally at the start of the interviews.

Semi‐structured one‐to‐one interviews were conducted by RB between December 2023 and March 2024. The interview schedule (see File S1) was informed by previous literature, the ABC‐X model (i.e. stressors, resources, perceptions, crisis/coping; Hill, 1958), the clinical experiences of the researchers and the siblings with lived/living experience, as above. Siblings were initially asked some questions about their interests and mapped out their family tree to warm up; they were then asked questions about their experiences as a sibling; and finally, they were asked what might have been helpful/what could be improved about the ward's sibling support. At the end of the interview, siblings were debriefed and signposted to relevant sibling and well‐being support.

All interviews were conducted remotely; eight via videoconferencing and two via telephone calls. Interviews were between 33 and 58 minutes in length (mean = 46.50, standard deviation = 7.71).

### Data analysis

Interviews were audio‐recorded and stored on an encrypted device and transcribed verbatim, with identifiable information removed prior to analysis. To address Aim 1, inductive reflexive thematic analysis was used, due to its flexibility and common use for exploring themes and patterns in experiential data (Braun & Clarke, 2006, 2019). Inductive coding with an experiential approach was taken. This enabled the experiences, meanings and the reality of siblings to be accessed and unanticipated themes rooted in the data to be explored.

The analysis phases described by Braun and Clarke (2006, 2019) were followed: (1) familiarization with the data by reading and re‐reading the transcripts, (2) generating codes, (3) constructing themes, (4) reviewing potential themes, (5) defining and naming themes and (6) producing the report. RB identified initial potential themes and subthemes. These were then reviewed by a second coder (EH), who checked that they reflected the semantic content of the dataset. Any remaining inconsistencies were discussed with a project supervisor (ALD), who had also read through and checked all the transcripts and codes and was involved in discussions about the themes and subthemes over multiple meetings. Following this process, themes and subthemes, and their interrelated links, were then consolidated and defined. Data analysis was conducted using NVivo for Mac (Version 14.23.2; QSR International Pty Ltd., 2023). As data saturation is not an element of reflexive thematic analysis (Braun & Clarke, 2021), we were informed by information power (Malterud et al.,^2016^).

After finalizing the thematic framework, in May 2024 the findings were presented to staff on the ward and in the wider oncology team. To address Aim 2, the findings were reviewed in consultation with the staff and used to identify pragmatic/feasible support recommendations, organized using the PPPHM (Kazak, 2006) (see File S2 for staff consultation topic guide). Aligned with the wider hospital, the PPPHM is already being worked towards by psychologists within other paediatric specialities.

### Author positionality

The researchers (authors) have a mix of clinical and research experience supporting CYP with serious physical illnesses and mental health difficulties and their families. All authors had no prior relationship with the participants. None of the authors have personal/lived experience of having a sibling with paediatric cancer. Professionally, all authors work within the field of psychology; one as an undergraduate psychology student, one as a trainee clinical psychologist and three as qualified clinical psychologists. All authors have previous experience of conducting research with reflexive thematic analysis and position themselves as being passionate about equitable and accessible healthcare for CYP and their families.

To address potential researcher bias, including blind spots, preconceptions and positionality, bracketing exercises (e.g. reflective interviews with colleagues) (Tufford & Newman, 2012) and reflexive journaling (McGrath, 2021; Orange, 2016) were utilized. All authors paid careful attention and regularly reflected on how their perspectives, research interests and experiences might be influencing the process of analysis, interpretation and identification of the siblings' needs and service improvements.

## RESULTS

An overarching narrative was found of siblings wanting to feel part of the cancer journey including their family's experience on the ward. Ultimately, siblings sought a sense of belonging, pertaining to involvement and closeness. Such desires to be more involved were woven throughout the main themes and their subthemes. Specifically, three main themes were found describing the experiences and perceived needs of siblings: (i) “what about me?”: overlooked and unseen, (ii) “always changing, never knowing”: the challenge of uncertainty and (iii) “let me be part of it all”: togetherness, communication and connection.

**FIGURE 1:**
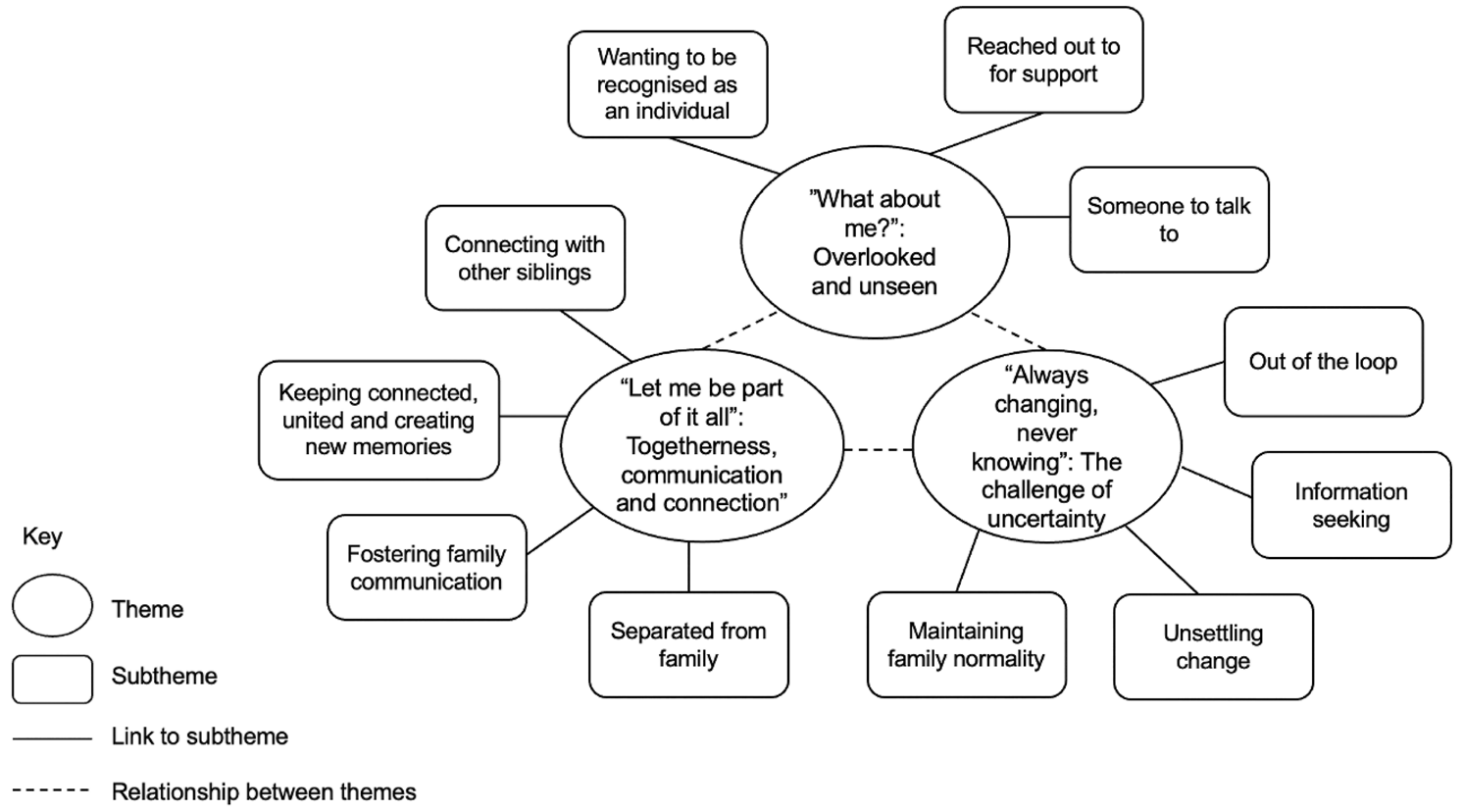
Overview of Themes and Subthemes.

Themes and subthemes are outlined in Figure 1 and summarized below with quotes which have been anonymized to protect confidentiality. In line with thematic analysis guidelines, theme and subtheme frequencies are not reported; instead, guidelines for quantifying language were followed, with ‘all’ referring to all siblings (or all but one), ‘most’ referring to more than half and ‘some’ referring to fewer than half but more than two (Hill et al., 2005).

### Theme 1: “What about me?”: Overlooked and unseen

This theme represents siblings feeling invisible and wanting to be reached out to for support. There was a sense of siblings wanting to be included and acknowledged in the context of their sibling's care, whilst also being seen and appreciated as themselves with an identity outside of their sibling's illness. This included having someone in the know outside of the family to talk to, just for them.

Most siblings emphasized wanting to be recognized as an individual. Siblings experienced a tension between understanding their unwell sibling needed more attention whilst also managing the challenges of feeling “*a bit forgotten*” (Adolescent 5) and “*spoken through rather than belonging*” (Adolescent 10) when staff were having conversations with their family. Siblings wanted ward staff to know they existed and notice them when they visited, “*maybe…said hello and stuff, might have made me feel like I mattered too…*” (Adolescent 4). Siblings wanted to feel appreciated as a “*person too*” (Adolescent 2), and be asked about other aspects of their life, by both ward staff and other people in their life, rather than just being known as ‘[name's] sibling’. Siblings valued areas of their life that did not change and experiences of not being treated differently, for example at school and within friendship groups, “*I liked that time with my friends was similar to before*” (Adolescent 4).

Siblings described a preference of being reached out to for support. They appreciated being made aware of support for siblings, as they valued siblings being “*thought of*” (Adolescent 7) during the hospital stay. Most siblings hoped “*someone would notice that I was actually finding it sort of hard*” (Adolescent 5) and check in with them proactively, rather than needing to go through their parents to access help.

I never asked for help, but I think if someone had asked me how I was doing, like actually doing honestly, then maybe then I could have got help…rather than needing to ask my parents to see if that was a thing… (Adolescent 5).

As such, most siblings found it helpful to have, or wanted to have, someone to talk to about their feelings and experiences. Among those who had someone to talk to, this included a school counsellor, a charity counsellor, a nurse on the ward and friends. Most siblings stressed it was important that this person knew the situation, whilst being “*someone separate from the family*” (Adolescent 6). Siblings emphasized that having this support, just for them, could help them to develop coping strategies and foster feelings of kindness and compassion towards themselves through feeling seen and validated.

Knowing that they were supporting me, as in specifically there for me, could help me to be less hard on myself and show me that my feelings matter too…siblings, we matter too. (Adolescent 10).

### Theme 2: “Always changing, never knowing”: The challenge of uncertainty

This theme describes the challenge of uncertainty faced by siblings, due to not knowing about their sibling's health and the ward and the varying changes in their lives. As such, siblings wished to have information shared with them and maintain some normality as a family.

Seeming out of the loop about cancer and treatment compared with the rest of their family, alluding to not being involved in cancer‐related conversations, was highlighted as challenging by most siblings; “*no one told me they were going into that hospital…so was a shock and confusing*” (Adolescent 1). There was a sense that “*parents would tell us less, but I think it made us worry more*” (Adolescent 5). Lacking information, some siblings assumed the worst outcomes, based on their limited experience and understanding of cancer and hospitals; for example, “*I just thought they would die even though that was actually not likely at all*” (Adolescent 3); “*I was scared for the worst or that my family would feel a bit broken forever*” (Adolescent 9); and *“I was worried she would die because when my grandad went to hospital, he then died…*” (Adolescent 4).

Consequently, most siblings described information seeking. This included wanting to “*know what was happening*” (Adolescent 3) *“about the cancer*” (Adolescent 6), including the treatment, hospitalization and prognosis. Such information could help to reduce assumptions by helping “*me to stop imagining stuff or the worst outcomes*” (Adolescent 5) and know “*what to say to others*” (Adolescent 9) when asked about their unwell sibling. One sibling found a book “*about having a brother or sister with cancer*” (Adolescent 7) helpful. Some siblings reflected “*it got easier with time because of knowing what was happening a bit more”* (Adolescent 8). Most siblings also wanted more information about the ward, such as what their room looked like, the staff caring for their sibling and who they could ask their questions to. Suggestions for providing this information included photos, a video such as a virtual tour to help visualize their family there, “*pictures of the ward so we could imagine it before we went there, would have been comforting to know where they were*” (Adolescent 6), and as such feel closer to them. It was noted that information needed to be developmentally appropriate; “*it was hard to understand the words in the letter mum got about it [the ward] so it being written for kids or teenagers like me*” (Adolescent 5). Once they had seen the ward, such benefits were identified, especially as the ward was often described as pleasant and the staff as friendly.

Experiencing unsettling change was described by most siblings. This included changes in routines, the atmosphere at home and seeing their sibling unwell with medical equipment and family members upset for the first time; for example, “*I remember most, vividly…when my dad came home and was crying because I don't think I had seen him cry until then…*” (Adolescent 6). For most siblings, responsibilities increased, including getting themselves ready for school, supporting other family members (e.g. younger siblings) and helping more at home. Some described building qualities such as independence and confidence from this, but also noted being “*too sad to be independent*” (Adolescent 4) and there being “*better ways to become more confident and independent than having a sibling be ill…*” (Adolescent 7). One sibling expressed a change in responsibility in relation to looking after their unwell siblings less; “*I helped to look after them a bit at home, so it was hard to lose that when they were in hospital…I couldn't be helpful in that way*” (Adolescent 4), demonstrating the complexities of role adjustment. For most, changes seemed ongoing; “*so it was a bit over the place, I sometimes didn't know who was staying that night until the day*” (Adolescent 5) and had adverse impacts in other areas of life such as keeping up with school expectations. Some siblings found new routines, helping them to adjust to a “*different normal*” (Adolescent 10) over time.

Among the uncertainty and change, maintaining family normality was described as helpful. Siblings appreciated their parents trying to keep things normal for them, such as engaging in usual family activities; for example, “*my mum and dad tried to keep things like normal sort of thing for me as much as they could because I told them the changes were kind of stressful*” (Adolescent 7) and “*I think it [playing board games] made it feel normal…yeah…and like it was OK and we were back in our home…even if we weren't…*” (Adolescent 2). Siblings appreciated when their unwell sibling's area on the ward could be decorated with some “*things from their room*” (Adolescent 10) to help make the space feel more homely and familiar.

### Theme 3: “Let me be part of it all”: Togetherness, communication and connection

This theme encompasses the narratives that focus on siblings desiring a sense of belonging within their family and peer group, the impacts of siblings being physically and emotionally apart from their families and ways of being brought closer as a family and to other siblings.

All siblings recalled being physically and emotionally separated from family, often feeling “*quite distant from my parents and [unwell sibling's name] because I couldn't see them as much…*” (Adolescent 4). Siblings expressed sadness, loneliness and longing for their family as a result; “*every day I did miss them*” (Adolescent 6). Some siblings stayed in other people's houses during their sibling's hospital stay which sometimes added to the physical distance from the hospital and meant seeing their family even less.

I think mostly being away from them, it would bubble up inside of me, the sadness. Like grow but I didn't know what to do with that. (Adolescent 1).

Given such separation, fostering family communication was perceived as helpful, rather than all keeping quiet. Some siblings thought parental support could be beneficial to help their parents have their emotional needs met and *“to explain to us [siblings] what was happening”* (Adolescent 2). Some siblings reported wanting family support, to make sure everyone was included and for *“us all talk to each other…and…helping my family to see what we were doing well and to make us believe we could get through it*” (Adolescent 1).

Similarly, siblings perceived keeping connected, united and creating new memories as important and helpful. This often meant being together in different ways; for example, “…*sometimes we'd wait to know [the food on the ward] and then we would try to have the same, so we felt together*…” (Adolescent 5) and “*we wore the same bracelets to feel closer*” (Adolescent 10). Some siblings described “*appreciating each other more*” and feeling more united regarding “*us all against the cancer, together*” (Adolescent 7). There was a sense of feeling “*closer in my heart*” (Adolescent 7), even if in distance they were further apart. Some siblings spent more quality time with certain family members; for example, “*I saw my dad and then my aunt a bit more actually because they came to stay at the house…it was nice doing things with them*” (Adolescent 9). Still making family memories during this time was also valued, such as “*I got a hot chocolate downstairs from the [hospital] café which was good…my dad said as a treat*…” (Adolescent 7).

Alongside keeping connected with their family, all siblings discussed a desire to connect with other siblings on the ward who “*get it*” (Adolescent 7) and could help them make sense of the experience. As part of this, siblings who had sibling(s) other than their unwell one found connecting with them beneficial; “*…talking to other siblings too…because having my other brother was really helpful*” (Adolescent 6). Most siblings specified wanting to connect with other adolescent siblings, to feel they *“belonged, as younger children would not understand the challenges of being our age as much…”* (Adolescent 10). A variety of modalities were suggested, including *“having things that siblings would say to other siblings, make them feel less alone*” (Adolescent 4) and “*doing activities whilst talking about hard things and learning ways of coping*” (Adolescent 10).

### Recommendations for improving sibling support

The findings within the themes/subthemes were reviewed in consultation with staff and used to identify pragmatic/feasible recommendations for improving sibling support, mapped onto the PPPHM (Kazak, 2006).

**FIGURE 2:**
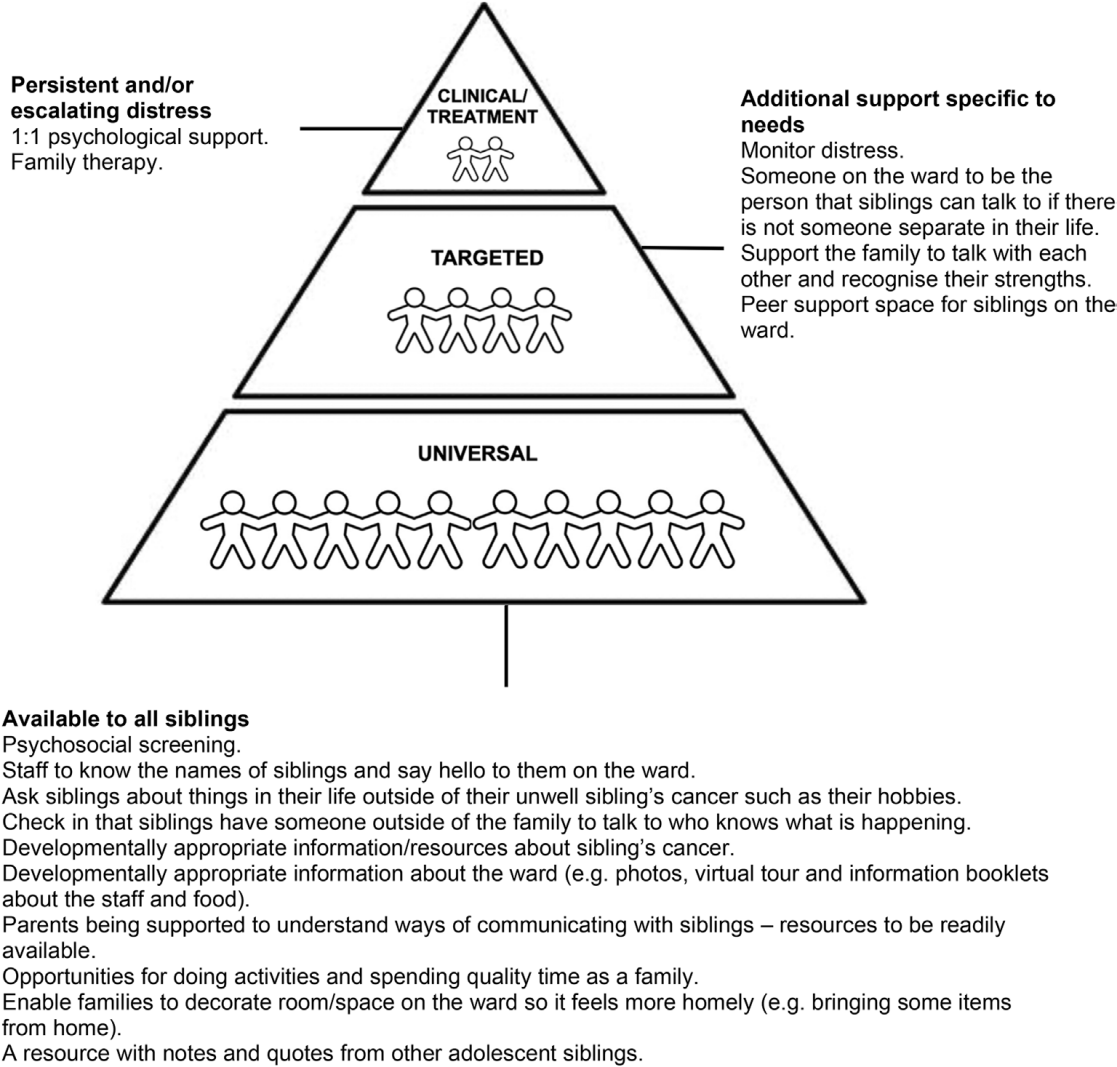
Summary of Service Improvement Recommendations Organized Using the Paediatric Psychosocial Preventative Health Model (Kazak, 2006).

These recommendations are displayed in Figure 2, showing the universal support recommended for all siblings and then the more targeted and clinical/treatment support for siblings requiring more intensive support.

## DISCUSSION

This project sought to understand the experiences and perceived needs of adolescent siblings of paediatric oncology inpatients to inform service improvement recommendations for sibling support. Key themes were found, all interconnected by an overarching narrative of wanting to feel part of the cancer journey including their family's experience on the ward. Such findings align with the family‐centred approach to paediatric inpatient care, as recommended by NICE (2021) guidelines, whereby care is planned in partnership with and around the needs of the whole family.

Consistent with the evidence base, siblings described an array of emotions, including anxiety, sadness, shock and loneliness (Løkkeberg et al., 2020; Weiner & Woodley, 2018). Developing qualities, such as confidence and independence, were highlighted by some siblings, although siblings described desiring that they had built them in ways other than having an unwell sibling. Whilst understanding that their unwell sibling required support, siblings perceived themselves as being overlooked, as previously noted (Toft et al., 2019; van Schoors et al., 2019). As well as included in the care, siblings also wanted to be recognized as individuals, rather than just their sibling's sibling. The need for both belonging and individual appreciation is perhaps particularly pertinent for this age group, as adolescence is a time of seeking autonomy and independence to carve out personal identity whilst also consolidating social identity (Klimstra & van Doeselaar, 2017; Meeus, 2018).

A proactive approach for sibling support was desired, rather than needing to seek support themselves or go through parents. This is important, given that reluctance to disclose and low parental awareness have been recognized as barriers to siblings accessing support (Brosnan et al., 2022; Weiner & Woodley, 2018). Being thought of by ward staff was also valued and may be beneficial, as having trusted relationships with relevant adults has been shown to facilitate help‐seeking among adolescents (Aguirre Velasco et al., 2020). To identify siblings experiencing psychosocial difficulties to then triage for support as appropriate, families have supported regular psychosocial screening including via electronic questionnaires to support feasibility (Deatrick et al., 2022; Long et al., 2017). This may include the ‘Psychosocial Assessment Tool’ (Kazak et al., 2018), a parent‐reported screener based on the three‐tier PPPHM (Kazak, 2006) or the newly developed ’PAT Sibling Modules’ with validated diagnosis and follow‐up versions which assess sibling risk factors and reactions respectively (Davis, Alderfer, et al., 2023; Davis, Brosnan, et al., 2023; Long et al., 2022). Utilizing the ‘Sibling Cancer Needs Instrument’ (SCNI) (Patterson et al., 2014), a sibling‐report measure which specifically assesses the unmet needs of adolescents and young adults with a sibling with cancer, may also be beneficial. When experiencing difficulties, having someone to talk to outside of the family (e.g. one‐to‐one) was also reported as beneficial. This may include having a space to talk about the impact of the illness on their life and adjustment (Gerhardt et al., 2015; Wiener et al., 2015).

As alluded to in the current study, lack of cancer‐related information (including specific to their sibling) has been identified as a common unfulfilled need among adolescent siblings (Masoudifar et al., 2022) and linked to assumptions surrounding potential prognoses and therefore distress (Weiner & Woodley, 2018). Having access to developmentally appropriate informational resources about cancer in general and specific to their sibling could address such assumptions and reduce distress through having a more informed perception of the situation.

Previous research has indicated that most siblings do not seek information outside of their known network, preferring it to come from their parents or other trusted people (van Schoors et al., 2019; Wawrzynski et al., 2023). Indeed, siblings in the current study appreciated being informed by staff and their family. As such, the sibling psychosocial standard of care in the standards for the psychosocial care of children with cancer and their families proposed by Wiener et al. (2015) recommends that parents and staff should be supported to facilitate their communication with siblings. Family barriers to information acquisition were reported, including reluctance from parents to share information (out of protection) and siblings to seek information or ask for help from their family (to not take the focus away from their unwell sibling) (Brosnan et al., 2022; Ilic et al., 2023). Such findings further support utilizing a family‐centred approach to involve siblings, for example, parental and family support for communication (e.g. information sharing, recognizing strengths), to help foster the perception that they could get through it together. Previous research has also identified a lack of knowledge of issues faced by siblings among staff as a barrier (Gerhardt et al., 2015), indicating staff training may also be beneficial.

In line with previous research, siblings described unsettling changes, including to their routines and responsibilities (Løkkeberg et al., 2020; van Schoors et al., 2019; Weiner & Woodley, 2018), making it harder to uphold expectations in other areas of their lives such as school (Tsimicalis et al., 2018). Such changes may be compounded by the context of their psychosocial stage of development, whereby adolescents strive to create a continuous sense of identity as opposed to role confusion (Erikson, 1950). As such, maintaining some perception of normality (Yang et al., 2016), both within the family and their wider life as individuals, was desired and experienced as helpful.

Consistent with the current evidence base, separation from family was difficult among siblings (Yang et al., 2016). During this time, perceived togetherness and connection with family was viewed as important. Better family functioning (e.g. cohesion) has been associated with lower levels of sibling loneliness and better psychosocial adjustment outcomes (van Schoors et al., 2016, 2021). Siblings found benefits from spending quality time and making new memories together with their families, something that ward staff could help to facilitate.

Connecting with other siblings was desired, particularly of similar age who could understand the experience of having a sibling with cancer in hospital in the context of other typical adolescent stressors. Seeking such connections may be especially pertinent during adolescence, a developmental period when peer groups become increasingly important (Meeus, 2018; Ragelienė, 2016). Benefits from group‐based interventions for siblings of CYP with cancer have been identified, including improvements in psychosocial adjustment, knowledge of cancer, sense of belonging, identity and family relationships (Guan et al., 2021; Mooney‐Doyle et al., 2021). Such peer support spaces have also been desired as places to improve their coping skills and learn information through activities and workshops (Ilic et al., 2023). As such, resources fostering peer support could also be helpful for siblings of inpatients.

### Clinical implications for sibling support

Informed by this project's findings, recommendations for improving sibling support were identified. Based on their experiences, such recommendations could support siblings during the hospital admission (i.e. the stressor) with their coping resources (e.g. developmentally appropriate information, communication, family and peer support) and perceptions (e.g. being/feeling seen, reducing assumptions, maintaining normality, identifying family strengths) of being able to manage, therefore helping to reduce risks of poor psychosocial adjustment (Hill, 1958).

The recommendations proposed in the current study, organized using the PPPHM (Kazak, 2006), complement existing standards and guidelines for the psychosocial care of siblings in paediatric oncology (Gerhardt et al., 2015; NICE, 2014; Wiener et al., 2015). For example, the standards of care (Gerhardt et al., 2015; Wiener et al., 2015) for siblings specify the need for providing appropriate support to those who are psychosocially at risk. Furthermore, our recommendations align with the recently published ‘Sibling Services Blueprint’ for systematic psychosocial screening and support for siblings by (Davis et al., 2024). Also organized using the PPPHM (Kazak, 2006), this blueprint includes routine use of psychosocial screening tools, universal‐level support for all siblings (e.g. educational materials), awareness of and commitment to addressing sibling needs (e.g. onwards) and a range of options for sibling psychosocial support matched to the level of psychosocial need.

### Strengths and limitations

To our knowledge, this was the first study to investigate the experiences and perceived needs of adolescent siblings on a paediatric oncology ward in the UK. This study developed several service improvement recommendations for better‐supporting siblings. However, siblings seeking support and involvement may have been more likely to take part, perhaps influencing some of the findings. Although most siblings had visited their sibling with cancer on the ward, data on the frequency and length of visits were not collected. Moreover, whilst the sample of siblings was diverse in some respects (e.g. gender), most were from nuclear families, most were White British and all were English speaking. Therefore, it is difficult to ascertain whether such findings are generalisable to all siblings who met the project's inclusion criteria.

### Future directions

Given the eligibility criteria, which importantly enabled an in‐depth understanding to be gathered for the group of interest, the findings may not apply to non‐adolescent and bereaved siblings. Future projects focusing on other sibling groups could help to establish whether experiences and needs may vary for those siblings. The next critical steps are the implementation and evaluation of the recommendations to inform evidence‐based care.

Although this project was specifically for the ward being studied, it is likely relevant to other similar contexts at least in the United Kingdom. Nevertheless, exploring the experiences and needs of siblings in other paediatric oncology wards may also be useful, given that unmet support needs among siblings are a wide‐scale issue (Davis, Alderfer, et al., 2023; Davis, Brosnan, et al., 2023; Yang et al., 2016). This may include building collaborations with academic partners to harness innovative approaches, such as longitudinal designs to investigate the trajectory of experiences and understand the most optimal timepoints for providing sibling support.

## CONCLUSION

This qualitative study captured the lived experiences and needs of adolescent siblings of CYP in a paediatric oncology ward. The overarching narrative found that siblings wanted to feel part of the cancer journey including their family's experience on the ward. Interconnected by such desires to be more involved, three key themes were found. These findings informed recommendations for sibling support, organized with ward and oncology staff using the PPPHM (Kazak, 2006). Based on sibling experiences, it is considered that such recommendations could support their resources and perceptions for coping, therefore helping to reduce risks of poor psychosocial adjustment (Hill, 1958). Implementation and ongoing evaluation are needed, as well as exploring the experiences of all siblings, to help ensure sibling support is continually improved and their needs are met.

## AUTHOR CONTRIBUTIONS

**Rachel Batchelor:** Conceptualization; formal analysis; investigation; methodology; project administration; resources; visualization; writing – review and editing; writing – original draft; data curation. **Matthew Hotton:** Conceptualization; methodology; project administration; resources; supervision; writing – review and editing; writing – original draft. **Eloise Harris:** Formal analysis; investigation; project administration; writing – original draft; writing – review and editing. **Alex Lau‐Zhu:** Conceptualization; methodology; project administration; resources; writing – original draft; writing – review and editing; supervision. **Annabel L. David:** Conceptualization; methodology; project administration; supervision; resources; writing – original draft; writing – review and editing.

## CONFLICT OF INTEREST STATEMENT

The authors declare that they have no competing interests.

## Supporting information


File S1.

File S2.

## Data Availability

The data that support the findings of this study are available on request from the corresponding author. The data are not publicly available due to privacy or ethical restrictions.
