# Supplementary material for: “We are here too”: Experiences and perceived support needs of adolescent siblings of Paediatric oncology inpatients
Source: Br J Health Psychol. 2025 Feb 17;30(1):e12785. doi: 10.1111/bjhp.12785 (PMC11831875; doi:10.1111/bjhp.12785)
Supplement: Supplementary file 1 — File S1. [file BJHP-30-0-s001.docx]

**Supplementary File 1**

**Semi-Structured Interview Schedule**

**Project Title: Improving Support for Siblings of Children and Young People on [Ward Name]**

Project lead: Rachel Batchelor

*[To check that consent/assent forms have been returned prior to start of interview]*

**INTRODUCTION**

*[Parent/carer may be present for introduction – this is up to the sibling]*

Hello, my name is Rachel Batchelor. I am a Trainee Clinical Psychologist. Thank you for meeting with me today, I am really grateful.

As a reminder, the project that we are meeting for today aims to improve support for siblings of children and young people on [ward name].

When you were first told about this project, you and your parent(s)/carer(s) will have been sent information sheets and consent forms. Do you have any questions?

You and your parent(s)/carer(s) will have also been sent a short questionnaire to gather more information about you and [name] such as your ages. This gives us helpful background information about your family and may help us to think about how the service could be improved for siblings who may have things in common with you, or who may be different to you. Thank you for completing this and sending it back to us.

*[If they have not returned the short questionnaire, go through and complete it together- may need parent/carer to support with some questions]*

I understand that when a child or young person is in hospital it can impact their siblings. I am going to start off by asking some questions about what it is like to have a sibling on [ward name]. We will then talk about any ideas you have about what you might have found helpful whilst [name] was on [ward name].

In terms of **what to expect from our discussion**:

- I am not part of the team at [ward name]; I am just doing this project with the service.
- I will not tell the staff on [ward name] what you tell me, only a summary of all of the answers I get where they will not know who said what.
- All information from our discussion that could identify you such as names will be removed when I write up this project, to make sure your answers remain private.
- I understand that talking about these experiences may be hard, so please let me know if there are any questions that you do not want to answer.
- You can also take a break or stop the interview at any time.
- It is also OK if you are not sure about a question/answer.
- I am interested in both what the ward is doing well and what could be done differently for siblings. This information will hopefully help [ward name] to make changes to improve the support for other siblings in the future.

Whilst I have some questions for you, this is also your opportunity to tell me anything else about having a sibling on [ward name] that you think is important.

We will be talking for around 30 minutes to one hour.

Are you OK for your parent/carer to leave the room whilst we go through the questions together?

*[Parent/carer to leave room]*

Thank you. We are going to touch on some sensitive topics today, if that feels OK with you. Before we start, I **want to check in with how you are feeling today?**

Would it be helpful to do a **breathing/grounding exercise together**?

Choice of either 54321 technique or breathing exercise

**Check in again** after breathing/grounding exercise.

Before I begin asking the questions I have for you, do you have any questions you want to ask me?

*[Answer any questions the sibling may have]*

Are you OK for me to now start recording and ask you my questions?

*[Turn on recording]*

**WARM UP AND BACKGROUND QUESTIONS**

I would like to start with a question to find out a more about you. What sorts of things do you enjoy doing?

**Prompts:** What do you do in your free time? Do you have any particular hobbies or interests?

We are now going to think a bit about your family. To help us with this, I would like to draw out your family tree.

Led partly by the sibling’s answers, their family tree will be mapped out by discussing information such as their number of siblings, positions in family of interviewee and sibling with cancer, relationship to sibling (e.g. full sibling, half-sibling, stepsibling), who usually at home/in their household, etc.

*[Questions regarding the relationships in the family will also be asked during this exercise, such as those below]*

Who do you feel closest to in the family?

Who are you most like/similar to in the family?

How might your family describe you?

How would you describe [name]?

Who do you spend the most time with?

What do you enjoy doing with family members?

Who do you talk to when you are upset or need help?

**EXPERIENCES**

Now I am going to ask a bit more about your experiences as a sibling. Could you tell me what it was like having a sibling stay on [ward name]?

How did you feel when [name] was on [ward name]?

**Prompts:** *[prompt with emotions such as worry and sadness – and what these emotions were in relation to]*

What are your strongest memories from when [name] was on [ward name]?

What was most difficult or challenging about [name] being on [ward name]?

How was your homelife impacted when [name] was on [ward name]?

Where did you stay whilst [name] was on [ward name]?

How were other things impacted when [name] was on [ward name]?

**Prompts**: School life? Routines? Role/responsibilities? Anything else?

How about your relationships with your family, could you talk about how they may have changed or been affected when [name] was on [ward name]?

**Prompts:** *[make sure at least sibling and parent(s)/carer(s) are discussed, may consider time spent together, closeness, communication, missing them]*

Did you visit [name] whilst they were on [ward name]?

If yes:

What was your experience of visiting [name] whilst they were on [ward name]?

**Prompts**: What was the ward environment like for you? What was helpful? What was unhelpful?

What was it like seeing [name] on [ward name]?

If no:

Was there a reason why you did not visit [name] whilst they were on [ward name]?

**Prompt**: *[if wanted to visit but barrier identified]* Is there anything that would have made it feel possible or easier to visit them?

Interviewer: Is there anything that helped you or your family cope when [name] was on [ward name]?

**Prompts:** *[this may include self-care, personal/family strengths, personal/family beliefs, particular support]*

Were you aware of any support you could access while [name] were on [ward name]?

**Here the interview can progress in one of two ways. If yes, continue. If no, skip to improvements.*

What support was available?

Did you access any support?

If yes:

What did you access and what was your experience of the support?

**Prompts**: What was helpful? What was unhelpful?

If no:

Was there a reason why you did not access the support?

**Prompt**: *[if barrier identified]* Is there anything that would have made it feel possible or easier to access the support?

Is there anything that helped you or your family cope when [name] was on [ward name]?

**Prompts:** *[this may include self-care, personal/family strengths, personal/family beliefs, particular support]*

**IMPROVEMENTS**

Now I am going to ask about what could be improved about the support for siblings. What support would you have wanted whilst [name] was on [ward name]?

**Prompts**: *[explore a range of different support such as]* one-to-one support, peer support, family support, particular activities, resources, information booklet.

Is there anything you wish you had been told about [name] being on [ward name]?

**Prompts**: *[if sibling does not provide an answer]* About why they were on [ward name]? About the Ward itself? About their cancer or treatment?

What might it have been helpful for other people to have known or done?

**Prompts**: *[if sibling does not provide an answer]* Perhaps about how you were feeling or your experiences? Can explore in terms of parents, staff, teachers, friends, extended family and other people in life or community.

Is there anything that could have helped you or your family to cope better with [name] being on [ward name]?

Is there anything that could have helped you or your family to cope better when [name] first went onto [ward name] or when they went back home?

Is there anything that you would want to tell another child or young person who has a sibling on [ward name]?

**ENDING**

Before we end, is there anything that we have not talked about your experience of having a sibling on [ward name] that you would like to share with me, or think is important?

Were you expecting we would talk about anything that we have not?

Are we OK to end there or is there anything else to do with [ward name] that you would like to tell me?

Thank you very much for taking the time to talk to me today. We really appreciate you talking about your experiences so openly and you have been a great help to our project.

*[Turn off recording]*

*[Provide sibling and parent/carer (for siblings age under 16-years-old) debrief sheet. Allow time for any questions about the project]*

**DEBRIEF**

We have touched on some sensitive topics today – I wanted to check in with how you are

feeling?

Option for another breathing/grounding exercise.

Any questions?

Thank you again for taking part.
