# Supplementary material for: “We are here too”: Experiences and perceived support needs of adolescent siblings of Paediatric oncology inpatients
Source: Br J Health Psychol. 2025 Feb 17;30(1):e12785. doi: 10.1111/bjhp.12785 (PMC11831875; doi:10.1111/bjhp.12785)
Supplement: Supplementary file 2 — File S2. [file BJHP-30-0-s002.docx]

**Supplementary File 2**

**Staff Consultation Topic Guide**

**Section 1: Study Design and Findings**

Brief presentation of service improvement project’s methods and findings, including:

1. Rationale.
2. Recruitment and background data, such as demographic, family and cancer-related information.
3. Semi-structured interview topic guide.
4. Data analysis.
5. Thematic framework, with themes and sub-themes, derived from interviews (including example quotes).
6. Proposed recommendations (this part was held as a discussion), organised using the three-tier Pediatric Psychosocial Preventative Health Model (PPPHM; Kazak, 2006).

**Section 2: Staff Views**

Example questions to staff members:

1. How could Kamran’s Ward implement the proposed recommendations?
2. What further considerations are there regarding what is feasible for service implementation?
3. Are there any additional ideas for supporting siblings not mentioned in the thematic framework/recommendations?
